# Congenital Hypothyroidism and Bone Remodeling Cycle

**DOI:** 10.4274/jcrpe.3532

**Published:** 2017-06-01

**Authors:** Nazmi Mutlu Karakaş, Sibel Tulgar Kınık, Beril Özdemir, Nursel Muratoğlu Şahin, M. Ağah Tekindal, Ayşegül Haberal

**Affiliations:** 1 Başkent University Faculty of Medicine, Department of Pediatrics, Ankara, Turkey; 2 Başkent University Faculty of Medicine, Department of Pediatric Endocrinology, Ankara, Turkey; 3 Dr. Sami Ulus Obstetrics and Gynecology, Children’s Health and Disease Training and Research Hospital, Clinic of Pediatric Endocrinology, Ankara, Turkey; 4 İzmir University Faculty of Medicine, Department of Biostatistics, İzmir, Turkey; 5 Başkent University Faculty of Medicine, Department of Biochemistry, Ankara, Turkey

**Keywords:** congenital hypothyroidism, bone marker, thyroxin

## Abstract

**Objective:**

The present study aimed to evaluate the biochemical markers of bone turnover in children with congenital hypothyroidism during the course of treatment as compared to healthy children selected as controls.

**Methods:**

The study included 31 children with congenital hypothyroidism and 29 healthy children. In both groups, we evaluated serum procollagen type-1 N-terminal propeptide (PINP) and tartrate-resistant acid phosphatase type 5b isoform (TRACP 5b) levels as bone turnover markers.

**Results:**

In both groups, thyroid hormone levels were within normal limits. The levels of vitamin D were significantly higher in the cases with congenital hypothyroidism. Although PINP levels were not found to be different, TRACP 5b levels which are related to osteoclastic activities were significantly higher in the control group.

**Conclusion:**

We did not detect an increase in bone resorption in patients with congenital hypothyroidism, despite long-term treatment with LT4. Our results suggest that with effective vitamin D treatment and thyroxin replacement, congenital hypothyroidism is not a deleterious factor for bone turnover.

## What is already known on this topic?

Long-term treatment with LT4 increases bone resorption in patients with congenital hypothyroidism.

## What this study adds?

Our results suggest that with effective vitamin D and thyroxin replacement, congenital hypothyroidism is not a deleterious factor for bone turnover.

## INTRODUCTION

Congenital hypothyroidism (CH) is one of the most common preventable causes of intellectual disability (1). Effects of hypothyroidism have decreased in babies administered treatment in accordance with the neonatal screening program (2). Untreated CH results in delayed bone age, growth retardation, and short stature (3).

Thyroid hormones also have important effects on the bone remodeling cycle. However, this effect has been shown to increase in favor of bone resorption, especially in thyrotoxicosis (4). In some studies in adults, long-term treatment with levothyroxine has been reported to decrease bone density, which may result in bone fractures (4,5,6). In contrast, some studies have indicated that long-term treatment would not affect bone density (7,8). Both hypothyroidism and hyperthyroidism have been associated with an increased risk of fractures. It has been determined that remodeling increases in adults in the course of hypothyroidism; in this situation, osteoclastic duration is increased twofold, whereas osteoblastic duration is prolonged fourfold. These changes result in low bone turnover and an overall failure to gain bone mass and mineral (3).

In children diagnosed with CH in the neonatal period and treated for a long time, the bone remodeling cycle may indicate the effects of thyroid hormone treatment or those of CH. Additionally, the presence of vitamin D deficiency also affects the bone cycle (9). In the assessment of the bone cycle by serum analysis, the new bone formation may be evaluated by the level of serum procollagen type 1 N-terminal propeptide (PINP), and bone resorption by the level of the enzyme tartrate-resistant acid phosphatase type 5b isoform (TRACP 5b), which is secreted by osteoclasts (10). The present study aimed to evaluate the biochemical markers of bone turnover in children with CH who had been followed up during the course of treatment and in healthy children selected as controls.

## METHODS

The study included 31 children with CH followed up by the Başkent University, School of Medicine, Pediatric Endocrinology outpatient clinic and 29 healthy children followed up by the General Pediatrics outpatient clinic. All patients and their families were informed about the study, and consent was obtained from the families. Blood samples were withdrawn during the blood sampling for medical purposes.

Of the 31 patients with CH, 14 were cases of hypoplastic thyroid gland, one had agenesis, one had an ectopic thyroid gland, 14 had thyroid dyshormonogenesis, and one had a fetal goiter related to the TPO gene mutation. The initial LT4 treatment was administered at a dose of 7.5-15 µg/kg/day during the neonatal period.

The cases in the control group (n=29) were selected from those who had been followed up by the General Pediatrics outpatient clinic and had no chronic disease or abnormal neonatal screening result. They were not taking any medication. We did not question the dosage and duration of vitamin D intake in the two groups. 

Chronological age, weight, height, and percent of ideal weight for height were evaluated in all patients. All blood samples were withdrawn in the morning, at the same time. The serum levels of calcium, phosphorus, alkaline phosphatase (ALP), magnesium, thyroid-stimulating hormone (TSH), free thyroxine (fT_4_), parathyroid hormone (PTH), 25 hydroxycholecalciferol [25(OH)D3-Vitamin D], PINP, TRACP 5b, and urine calcium/creatinine ratio were analyzed in the biochemistry laboratory of Başkent University.

CH and control groups were compared according to vitamin D status, deficient or non-deficient.

Venous blood samples were drawn and sera were stored at -20 οC after centrifugation until testing. All assays were carried out at the same time. The levels of 25(OH)D were assayed using chemiluminescent microparticle immunoassay (Abbott Architect I2000 analyzer). The architect 25(OH)D assay is designed to have a limit of detection (LoD) of ≤10.0 ng/mL. Serum levels of calcium, phosphorus, ALP, magnesium, TSH, and fT_4_ were measured in the blood cell counter using an Abbott Cell-Dyn Ruby System (Abbott Diagnostic, Santa Clara, CA, USA).

Vitamin D levels at or below 15 ng/mL were defined as vitamin D deficiency (9).

PINP was measured by kit which is a sandwich enzyme immunoassay for in vitro quantitative measurement of PINP in human serum incubated for 30 minutes at 37 oC after covering it with the Plate sealer. The minimum detectable dose of PINP is typically less than 6.2 pg/mL. The intra- and inter-assay coefficients of variation (CV) reported by the manufacturer are <10% and <12% (Uscn Life Science Inc. Wuhan, Hubei, PRC).

TRACP was assayed by an immunoCapture Enzyme-Activity Assay in serum (BioVendor Research and Diagnostic Products, Czech Republic). In the BioVendor Human TRAP 5 Assay, calibrators, quality control and samples are incubated in microplate wells pre-coated with monoclonal anti-human TRAP 5 antibody. After a thorough wash, TRACP 5b bound to the antibody is allowed to react with the pNPP substrate at pH 5.5. The reaction is stopped by addition of hydroxine solution and absorbance of the resulting yellow color product is measured. The absorbance is proportional to the enzymatic activity of TRACP 5b. A calibration curve is constructed by plotting absorbance values against enzyme activities of recombinant TRACP 5 calibrators, and enzyme activity of unknown samples are determined (U/I) using this calibration curve. LoD is calculated from the real TRAP 5 values in wells and is 0.01 U/I. The intra- and inter-assay CVs reported by manufacturer are 2.4% and 7.6%.

This study was approved by the Başkent University Institutional Review Board and Ethics Committee (Project No: KA12/46) and supported by the Başkent University Research Fund.

### Statistical Analysis

Data were analyzed using the SPSS 20 (IBM Corp. Released 2011. IBM SPSS Statistics for Windows, Version 20.0. Armonk, NY: IBM Corp.) statistical software. Values were expressed as mean ± standard deviation, median (maximum-minimum), percentage, and frequency. Variables were evaluated after controlling the normality and homogeneity of variance prerequisites (Shapiro-Wilk and Levene’s tests). Data were compared between the two groups using the independent t-test (Student’s t-test); the Mann-Whitney U-test was used in the absence of prerequisites. Categorical data were analyzed using the Fisher’s exact test and chi-square test. When the expected frequencies were lower than 20%, evaluation by the “Monte Carlo simulation method” was performed in order to include these frequencies in the analysis. A p-value <0.05 was accepted as statistically significant.

## RESULTS

The characteristics of the cases with CH and control subjects are presented in Table 1. The values of chronological age, height-for-age, body weight, height, and percent of ideal weight for height were not different in the CH and control groups. Laboratory results on serum calcium, phosphorus, magnesium, ALP, fT4, TSH, PTH, and PINP levels were also not statistically different from one another and were also in the normal range. Mean TRACP 5b levels were significantly higher in the control group. The levels of vitamin D were significantly higher in the cases with CH (Table 1).

According to vitamin D status, TRACP 5b levels were significantly higher in controls. PINP levels were not found to be different (Table 2).

## DISCUSSION

The acid phosphatases are lysosomal enzymes, and the isoform 5b secreted from the osteoclasts is a marker used to detect bone resorption. On the other hand, procollagen type 1 N-terminal propeptide is a marker used for the evaluation of bone formation (11). In the present study, bone turnover was evaluated in children with and without CH.

Bone is a metabolically active tissue that undergoes continuous remodeling throughout life. In the course of bone remodeling, bone resorption is closely associated with new bone formation. Bone resorption and formation can be affected by some diseases and this process may be evaluated by the serum levels of several markers (10). The effect of congenital non-goiter hypothyroidism on bone is still not completely understood (12). Population studies have demonstrated that both hypothyroidism and hyperthyroidism affect bone remodeling and that they may increase the risk of bone fractures. Studies conducted on mice have shown that TSH affects the bone remodeling cycle negatively (4,5,6,7,8). Papadimitriou et al (13) determined that the low levels of TSH do not lead to bone loss in mice. In our study, TSH and fT^4^ levels were found to be similar in the hypothyroid and control groups, but the level of TRACP 5b, which is an indicator of bone resorption, was found to be lower in the hypothyroid group; in other words, bone resorption was low in hypothyroid cases.

Engler et al (14) showed that the thyroid hormones also affect bone turnover. In this study, the values indicating bone resorption were high in the cases with hyperthyroidism, before the administration of anti-thyroid treatment.

In several studies conducted on adults, TSH was reported to be suppressed by the administration of LT4 in patients with thyroid cancer and non-toxic goiter who did not have thyrotoxicosis, and bone density was reported to be decreased (4,5,6). In contrast to these results, Franklyn et al (7) and Marcocci et al (8) did not detect any change in bone density following treatment with LT4 for as long as 8 to 10 years. Leger et al (4) did not detect a change in bone mineral density in favor of bone resorption, in patients undergoing long-term LT4 treatment. Similarly, in our study, any effect on bone resorption has not been demonstrated in the group with CH.

Vitamin D contributes to bone turnover, and it is used as a supportive treatment, starting in infancy. We determined the level of vitamin D to be significantly higher in children with CH. When the cases were subdivided according to their vitamin D levels, TRACP 5b levels were found to be higher in the controls in both vitamin D sufficiency and deficiency situations. The value of PINP, which indicates the formation of new bone, did not differ significantly between the groups. In this study, we did not detect an increase in bone resorption in patients followed up in our clinic. The children with CH might have received more effective vitamin D replacement due to their close and more frequent follow-ups. The mean duration of follow-up in our hypothyroid patients was four years which may be considered as one of the limitations of the study. We did not question the duration and dose of vitamin D intake in the CH and control groups which is the other limitation.

Reference values for TRACP 5b have been published for Chinese in 2005 and subsequently for Caucasian children, in 2007 and 2012 (15,16,17). Rauchenzauner et al (16) has reported the TRACP 5b values for 50th percentile as 8.1 U/L for boys and 6.8 for girls in the 0-18 age group. Fischer et al (17) determined the TRACP 5b values for 50^th^ percentile at age 4 years to be 13.8 U/L for boys and 17 U/L for girls (17). The distribution of normal TRACP levels showed a wide range, depending on age, sex, and pubertal stage. Vitamin D levels were not given in the above studies.

Our study included an age-matched healthy prepubertal control group. We measured the vitamin D levels in both groups. We speculate that the main reason for the higher TRACP 5b levels in the controls might be the lower vitamin D levels.

In conclusion, we did not detect an increase in bone resorption in patients with CH, despite long-term treatment with LT4. Our results suggest that with effective vitamin D and thyroxin replacement, CH is not a deleterious factor for bone turnover.

## Figures and Tables

**Table 1:**
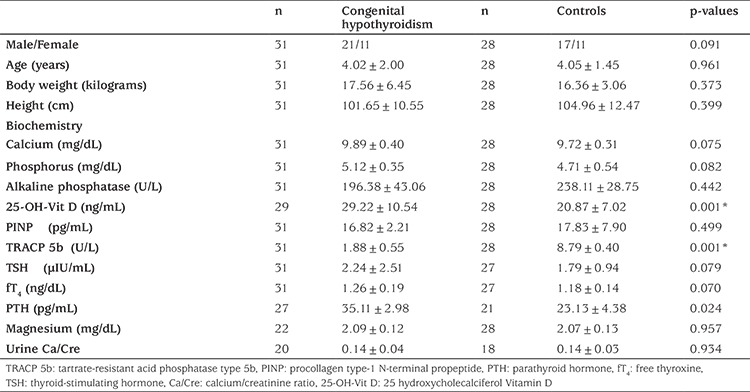
The characteristics of the cases with congenital hypothyroidism and the controls

**Table 2:**
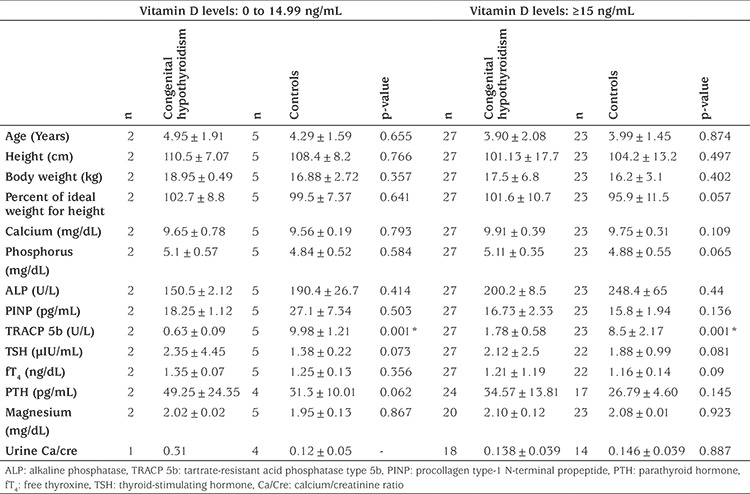
Comparison of the two groups according to vitamin D status
